# Perioperative inflammatory cytokines nursing screening test indicate the link between dysregulated lipid metabolism and reduced bone mineral density in obese osteoporosis patients: a retrospective study

**DOI:** 10.1186/s12891-026-09576-5

**Published:** 2026-02-03

**Authors:** Jingjing Wang, Yayi Shen, Xiaowei Lin, Yongxing Yang, Xiuqiang Lv, Xiexing Wu

**Affiliations:** Department of Orthopaedic Surgery, the First Affiliated Hospital, Suzhou Medical College, Soochow University, Suzhou, Jiangsu 215006 China

**Keywords:** Osteoporosis, Bone mineral density, Obesity, Lipid metabolism, Inflammatory cytokine

## Abstract

**Purpose:**

This study aims to investigate the underlying pathophysiological relationship between obesity and osteoporosis (OP) in obese individuals, involving lipid metabolism, inflammation, and bone mineral density (BMD).

**Methods:**

Data from 318 patients diagnosed OP at our hospital between January 2023 to December 2025 were collected and analyzed. The basic information of the patient included gender, age, BMI, drinking and smoking history, diabetes, hypertension and bone mineral density (T-scores) were recorded. Baseline peripheral blood was employed to calculate lipid markers and inflammatory cytokines. Linear regression and mediation analyses were employed to assess the relevance and differences.

**Results:**

Increased level of blood lipids and inflammatory cytokines were associated with increased risks of OP in obesity. Compared to normal-weight individuals, obese subjects exhibited significantly lower BMD. Dysregulated lipids (TC, TG, HDL-C, ApoB) negatively correlated with BMD in obesity. Pro-inflammatory cytokines (TNF-α, IL-1β, IL-6, IL-8) inversely associated with BMD, while anti-inflammatory IL-10 showed positive association. Hyperlipidemic obese individuals had elevated inflammatory cytokines (TNF-α, IL-1β) and exacerbated BMD loss. Mediation analysis revealed TNF-α mediated 41.91% and IL-6 mediated 33.20% of the TC-BMD association; TNF-α and IL-6 mediated 28.76% and 37.38% of HDL-C-BMD effects, respectively.

**Conclusions:**

Obesity-associated dyslipidemia drives BMD loss partly through inflammation-mediated pathways. Key inflammatory cytokines significantly mediate lipid metabolism’s impact on bone health. Targeting lipid-inflammatory crosstalk may optimize OP management in obese populations.

## Introduction

Osteoporosis (OP) represents a pervasive disorder of systemic bone metabolism, generally identified by decreased bone mineral density (BMD), disrupted bone microstructure, and dysregulated bone remodeling balance [1, 2]. This series of pathological physiological changes manifested clinically as bone pain while substantially increasing the risk of fragility fractures, particularly among elderly [2, 3]. The global prevalence of OP has been rising consistently each year, as highlighted in epidemiological studies, largely due to the aging of the population [2, 4]. Globally, osteoporosis is believed to impact nearly 200 million people. Following the age of 50, approximately 30% of women and 20% of men are predicted to suffer fractures connected to OP [5, 6]. The high frequency of fragility fractures and expanding healthcare burden have established OP as a critical public health challenge with profound societal implications [4, 7, 8]. Therefore, it is utmost importance to uncover new pathogenic factors and explore potential biomarkers for OP to improve fracture risk assessment and facilitate the development of early intervention strategies.

Obesity, as a prevalent multifactorial metabolic disorder characterized by excessive adipose tissue accumulation and aberrant fat distribution [9], has been widely demonstrates complex associations with degenerative musculoskeletal pathologies and altered bone metabolism [10, 11]. The traditional view posits that increased mechanical loading from body weight improves BMD and reduces fracture risk [12, 13]. However, whether obesity confers protection against osteoporosis and osteoporotic fracture remains contentious. While mechanical loading from body weight may enhance BMD, emerging evidence suggests obesity-induced metabolic dysfunction, including dyslipidemia and chronic inflammation, may counteract this benefit. Previous studies have confirmed that OP is a multifactorial chronic disease driven by several clinical risk factors besides age, sex and hormone secretion, notably a low body mass index (BMI) [14, 15]. Extensive clinical evidence consistently demonstrates a positive correlation between BMI and BMD among individuals with OP [16–18]. However, some studies indicate that obesity may negatively impact bone healing and exacerbate osteoporotic fracture risk via regulating osteoblastic and osteoclastic balance [19, 20]. The underlying mechanism may involve the excessive release of cytokines, which, due to the proinflammatory state associated with obesity, negatively affect bone health [21, 22].

Previous researches have highlighted a significant correlation between inflammation and OP [23, 24]. For example, recent research has shown that immune cells’ production of IL-9 and IL-10 can disturb immune-bone balance, directly contributing to inflammatory bone loss during the progression of OP [25, 26]. Research conducted in clinical settings has demonstrated that increased systemic inflammation is closely correlated with a higher likelihood of OP and fractures in individuals with frailty [27]. On the other hand, emerging research indicates that obesity-induced lipid dysregulation directly correlates with chronic inflammation [28], establishing a key pathophysiological link of various diseases including hypertension [29], diabetes [30], Alzheimer’s disease [31]. Nevertheless, there is a lack of focused research and clinical evidence to substantiate the link between obesity-related inflammation and OP.

Building on the theoretical framework outlined above, our study seeks to elucidate the pathophysiological link between obesity and OP through investigating the interaction among lipid metabolism, inflammation, and BMD.

## Materials and methods

This retrospective study was approved by the Ethical Review Committee of The First Affiliated Hospital of Soochow University (Approval No. 2026065) in compliance with the Declaration of Helsinki. Informed consent was formally waived by the Committee, as this research was classified as minimal-risk using de-identified retrospective data. To ensure data privacy and security: (1) All personal identifiers were irreversibly removed during data extraction; (2) Encoded study IDs were stored separately in password-protected hospital servers; (3) Analysis datasets contained only anonymized variables.

This study performed a retrospective analysis of 318 patients diagnosed with OP, all of whom received treatment at our hospital between January 2023 to December 2025. All cases met the following criteria: (1) OP diagnosis confirmed by BMD T-score ≤ -2.5 at lumbar spine or femoral neck; (2) without antiosteoporosis treatment before being hospitalized; (3) No long-term medication use affecting lipid metabolism; (4) exclusion of hyperthyroidism, hyperparathyroidism, malignant neoplasms, or any history of thyroid hormone/glucocorticoid administration; (5) patients without significant hereditary or congenital disorders affecting the skeletal or motor systems.; and (6) without complete paralysis and inability to walk were ruled out.

Data for this study were obtained from the participants’ electronic medical records. The BMI of each participant was measured by dividing their weight by the square of their height. A detailed assessment of the history of smoking/drinking history and diabetes/ hypertension status was also conducted. A complete laboratory evaluation was conducted for all patients using standardized assays at the First Affiliated Hospital of Soochow University, with blood specimens gathered on the second morning of hospitalization, after fasting overnight. The blood samples were collected using tubes containing 5 ml anticoagulated venous blood (EDTA). The tube was centrifugated at 3,000 rpm for 10 min. The tested blood lipid-related parameters, such as TG, TC, HDL-C, LDL-C, ApoA, ApoB and Lp(a), were determined by fully automatic biochemical analyzer (Beckman AU5800, USA). The concentrations of TNF-α, IFN-α, IFN-γ, IL-1β, IL-2, IL-4, IL-5, IL-6, IL-8, IL-10, IL-12 and IL-17 were measured using an enzyme-labelling measuring instrument (Liuyi WD-2102 A, China) with cytokine assay kits (Biolegend, USA), following the manufacturer’s instructions.

Statistical evaluations were performed using SPSS software version 26.0. The Kolmogorov-Smirnov test assessed the normality of the data. Results for data adhering to a normal distribution are reported as mean values with standard deviations, whereas skewed data are shown as medians accompanied by interquartile ranges. Differences among groups were tested with Student’s t-test and ANOVA for normally distributed variables, and with Mann-Whitney U or Kruskal-Wallis tests for data not meeting normality assumptions. To further examine potential correlation, mediation analysis was conducted using the SPSS PROCESS v4.2 to evaluate the role of inflammatory cytokines in the lipid-BMD relationship. We applied Model 4 (simple mediation) with 5,000 bootstrap iterations to estimate 95% bias-corrected confidence intervals. Mediation was considered significant if: (1) the direct effect was significant; (2) the indirect path had a 95% CI excluding zero. The proportion mediated was calculated as (indirect effect / total effect) × 100%. P-value blow 0.05 was deemed statistically significant (ns = not significant, *P < 0.05, **P < 0.01, ***P < 0.001).

## Results

### Baseline characteristics of study patients

This study included 318 participants after excluding those with medical interventions and those without data on blood lipids and inflammatory cytokines. Table 1 summarizes the characteristics of these participants. Participants were classified into three categories according to their BMI. Consistent with the National Health Commission of the People’s Republic of China criteria, BMI thresholds were 18.5–23.9 kg/m² (normal), 24.0–27.9 kg/m² (overweight), and ≥ 28.0 kg/m² (obesity). The average age of the participants was 68.67 ± 9.17 years, with 67.5% identifying as female. No statistically significant variations were observed among the groups in terms of their demographic or clinical profiles. However, the mean BMD value (T-scores) progressively increased the obesity group to the overweight group and subsequently to the normal weight group (3.12 ± 0.50 vs. −2.92 ± 0.40 vs. −2.81 ± 0.41, *p* < 0.001). Moreover, we compared lipid profile and inflammatory cytokines among the BMI subgroups in Table 2, revealing notable differences between normal weight and obesity group. Therefore, it is essential to further explore the correlation between obesity and the development of OP from the perspectives of lipid metabolism and inflammation. 

**Table 1:**
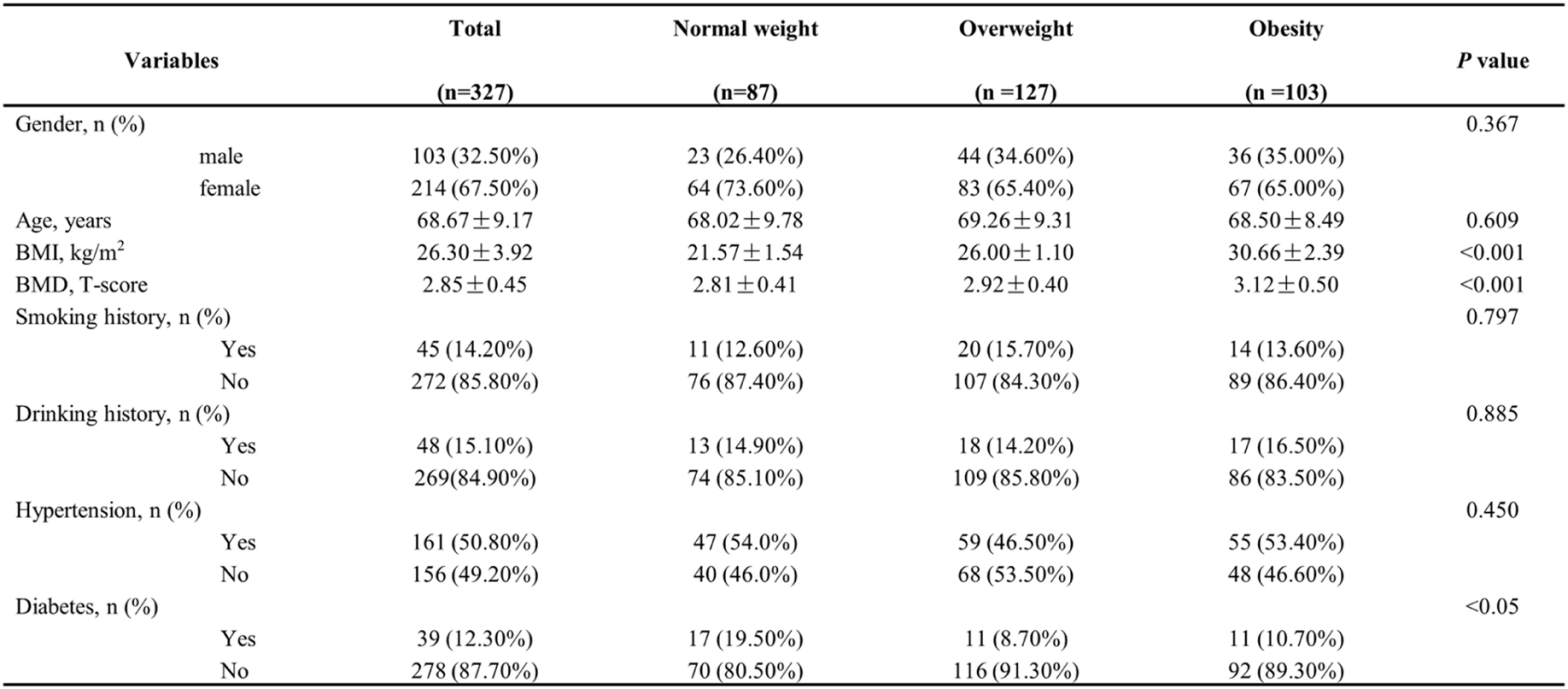
Baseline characteristics of the study participants with different BMI

**Table 2:**
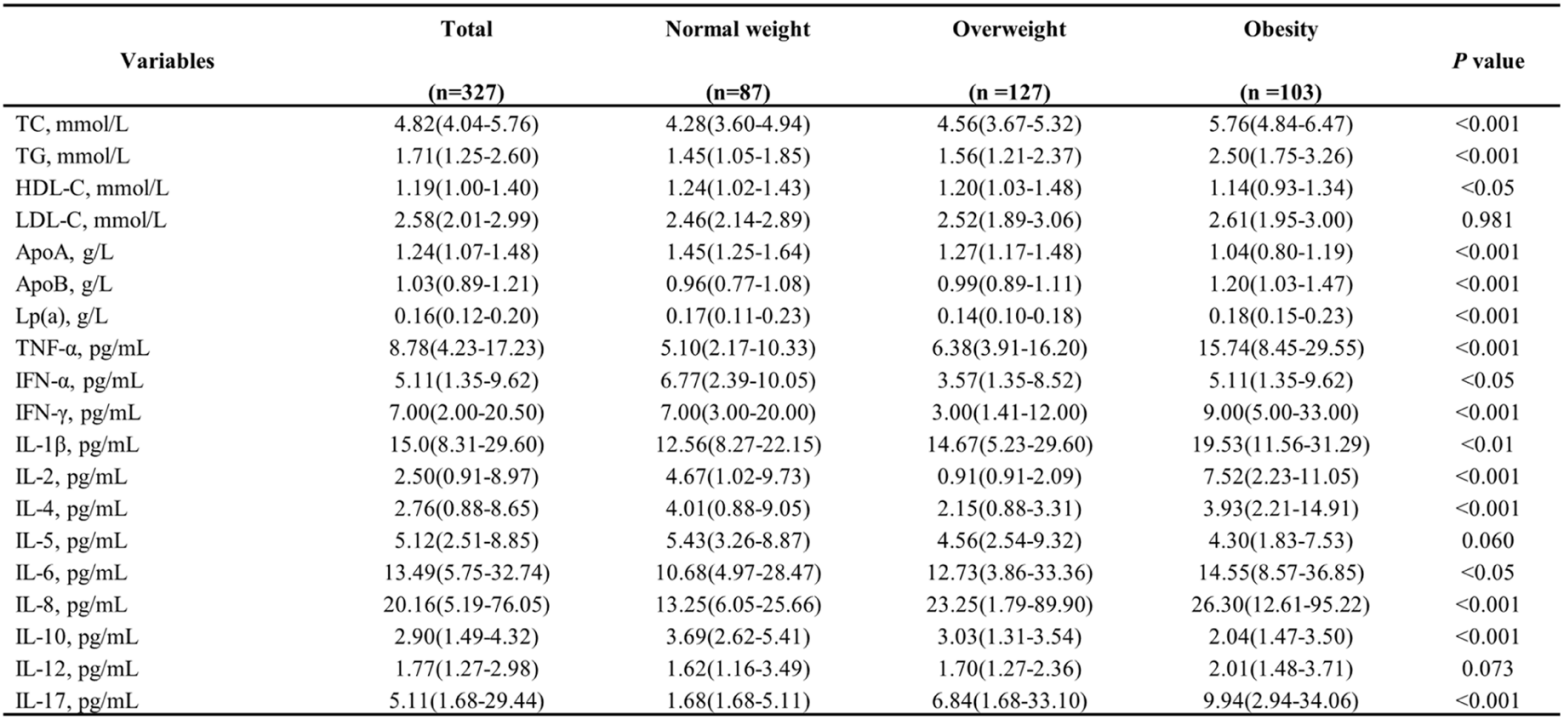
Baseline laboratory characteristics of the study cohort

*Abbreviations* *TC* Total cholesterol, *TG* Triglycerides, *HDL-C* High-density lipoprotein cholesterol, *LDL-C* Low-density lipoprotein cholesterol, *ApoA* Apolipoprotein A, *ApoB* Apolipoprotein B, *Lp(a)* Lipoprotein(a), *TNF* Tumor necrosis factor, *IFN* Interferon, *IL* Interleukin

### Associations of lipid profiles and inflammatory cytokines with BMD in obesity

Linear regression modeling was utilized to further explore the relationship among blood lipid, inflammatory cytokines, and lumbar BMD in patients with obesity (Table 3). In the basic model (Model 1), the results indicate that in obese patients, TG (β = -0.282; *p* < 0.001), TC (β = -0.241; *p* < 0.001), LDL-C (β = -0.824; *p* < 0.001), and ApoB (β = -0.327; *p* < 0.05), were negatively associated with BMD. After adjusting sequentially for demographic confounders and additional clinical covariates, these correlations continued statistically significant in Model 2 and Model 3. In the full adjusted model (Model 3), elevated TC (β = −0.297; *p* < 0.001), TG (β = −0.281; *p* < 0.001), and HDL-C (β = −0.79; *p* < 0.001) showed robust associations with BMD; and the correlation between ApoB and BMD was weakened (β = −0.312; *p* < 0.05). Among inflammatory cytokines, TNF-α (β = -0.005; *p* < 0.001), IL-1β (β = -0.005; *p* < 0.01), IL-6 (β = -0.005; *p* < 0.001), and IL-8 (β = -0.001; *p* < 0.01) an inverse correlation with BMD after adjusting for all covariates in the model (Model 3), while the anti-inflammatory cytokine IL-10 demonstrated a positive association (β = 0.041; *p* < 0.001).

**Table 3:**
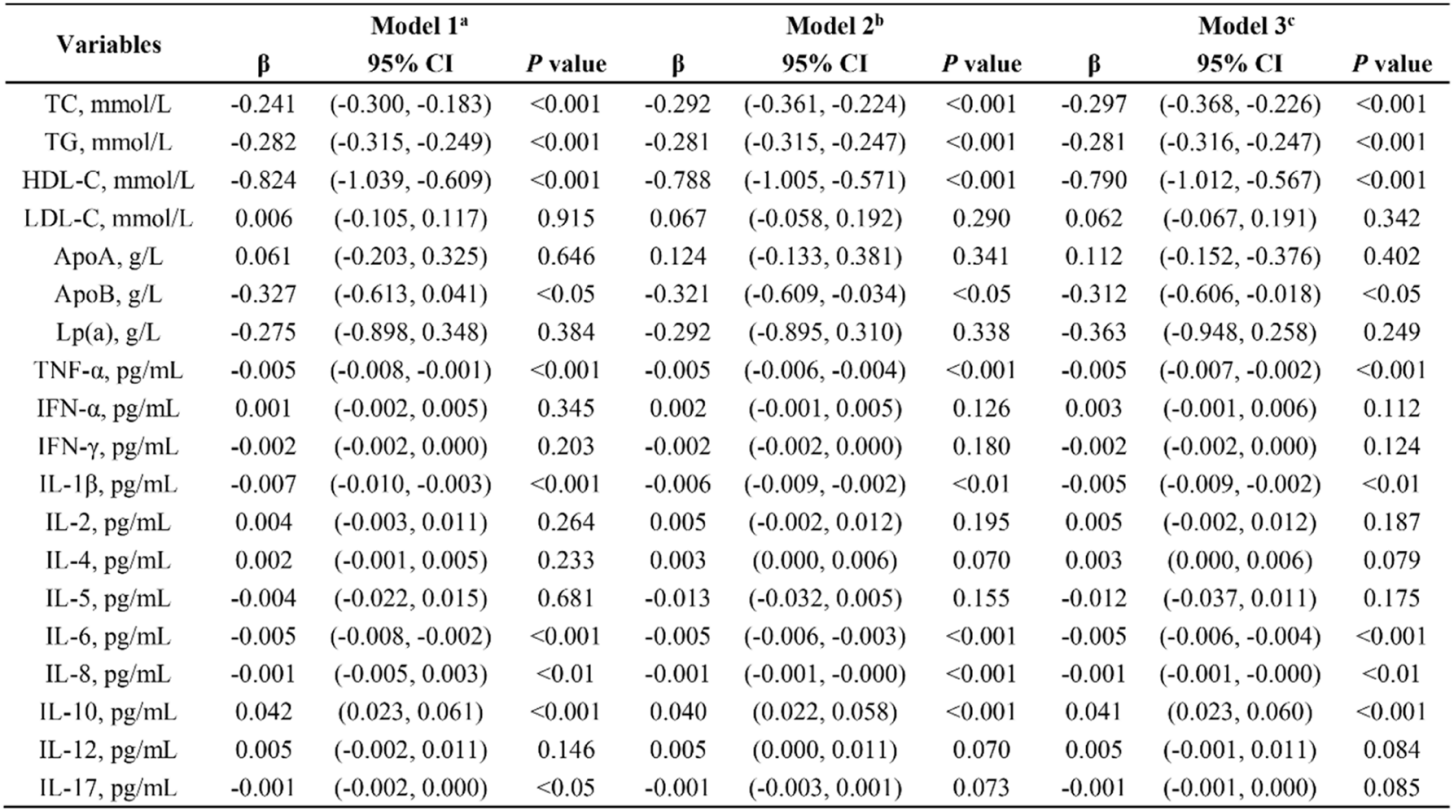
Comprehensive analysis of the associations between lipid metabolism, inflammatory cytokines, and BMD in the obese group

a Models were unadjusted

b Models were adjusted for sex, age, BMI

c Models were adjusted for sex, age, BMI, smoking and drinking alcohol history, hypertension, and diabetes *P < 0.05, **P < 0.01, ***P < 0.001

Table 4 presents the correlation analysis between lipid metabolism markers and inflammatory cytokines. In general, TNF-α exhibited a positive correlation with TC (*r* = 0.703, *p* < 0.001), TG (*r* = 0.556, *p* < 0.001), and HDL-C (*r* = 0.330, *p* < 0.001). levels. IL-8 also demonstrated a positively correlation with TC (*r* = 0.534, *p* < 0.001), TG (*r* = 0.578, *p* < 0.001), and HDL-C (*r* = 0.442, *p* < 0.01). In addition, IL-1β was positively correlated with TC (*r* = 0.342), TG (*r* = 0.311) and HDL-C (*r* = 0.283, all *p* < 0.001) levels, as well as with ApoB (*r* = 0.289, *p* < 0.01). As expected, the anti-inflammatory cytokine IL-10 is significantly negatively correlated with TC (*r* = -0.454), TG (*r* = -0.502), ApoB (*r* = -0.343, all *p* < 0.001) and HDL-C (*r* = 0.578, *p* < 0.01). Simultaneously, no significant correlations were found between IFN-α, IL-2, IL-4, and IL-12 with the lipid profiles, and Lp(a) demonstrated no significant correlations with any cytokine.

**Table 4:**
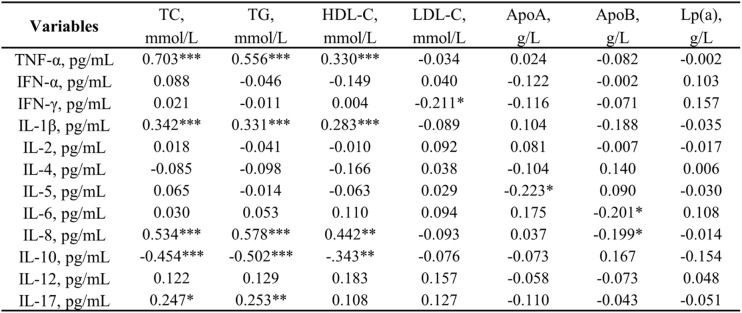
Correlation between lipid metabolism parameters and inflammatory cytokines in obese individuals

### Elevated inflammatory cytokines in obese subjects with hyperlipidemia

To assess the influence of blood lipids on inflammatory cytokines within the obesity cohort, the population was subdivided into groups based on hyperlipidemia status (Table 5). Through comparative analysis, we observed significant differences in TNF-α (H = 67.29, *p* < 0.001), IL-1β (H = 17.26, *p* < 0.01), and IL-8 (H = 16.70, *p* < 0.001) and IL-10 (H = 36.10, *p* < 0.001), between the groups. Specifically, hyperlipidemia robustly elevates TNF-α, IL-1β, and IL-8 levels in both normal-weight and obese individuals, while significantly decreased IL-10 levels in the obesity group. At the same time, IL-17 (H = 29.31, *p* < 0.001) only responded to obesity, showing no significant hyperlipidemia-associated changes in obesity subjects. IL-4 (H = 9.61, *p* < 0.05) significantly elevated in normal-weight with hyperlipidemia, but decreased in hyperlipidemic obese individuals. These findings highlight hyperlipidemia as a major driver of inflammatory cytokine secretion, with this effect substantially amplified in obesity.

**Table 5:**
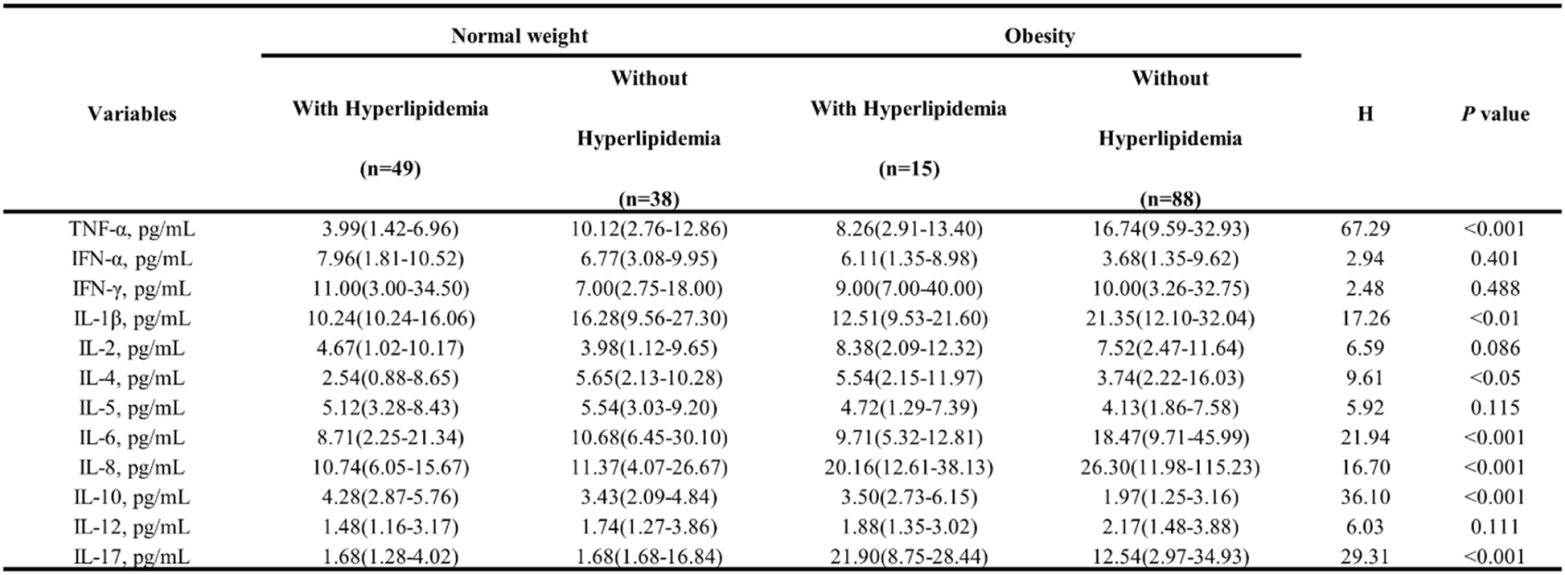
Comparative analysis of inflammatory cytokine levels among obese subgroups stratified by hyperlipidemia

Multiple linear regression analysis was used for a more detailed assessment of the association between inflammatory cytokines and BMD in patients, distinguishing those with and without hyperlipidemia (Table 6). Specifically, TNF-α (β = -0.688, *p* < 0.05), IL-1β (β = -0.425, *p* < 0.001), IL-6 (β = -0.659, *p* < 0.001), IL-8 (β = -0.455, *p* < 0.001), and IL-10 (β = 0.535, *p* < 0.001) showed statistically significant associations with BMD within the obese-with-hyperlipidemia subgroup. These inflammatory cytokine-BMD correlations were notably more pronounced in magnitude and statistical significance among hyperlipidemic obese individuals compared to non-hyperlipidemic obese group.

**Table 6:**
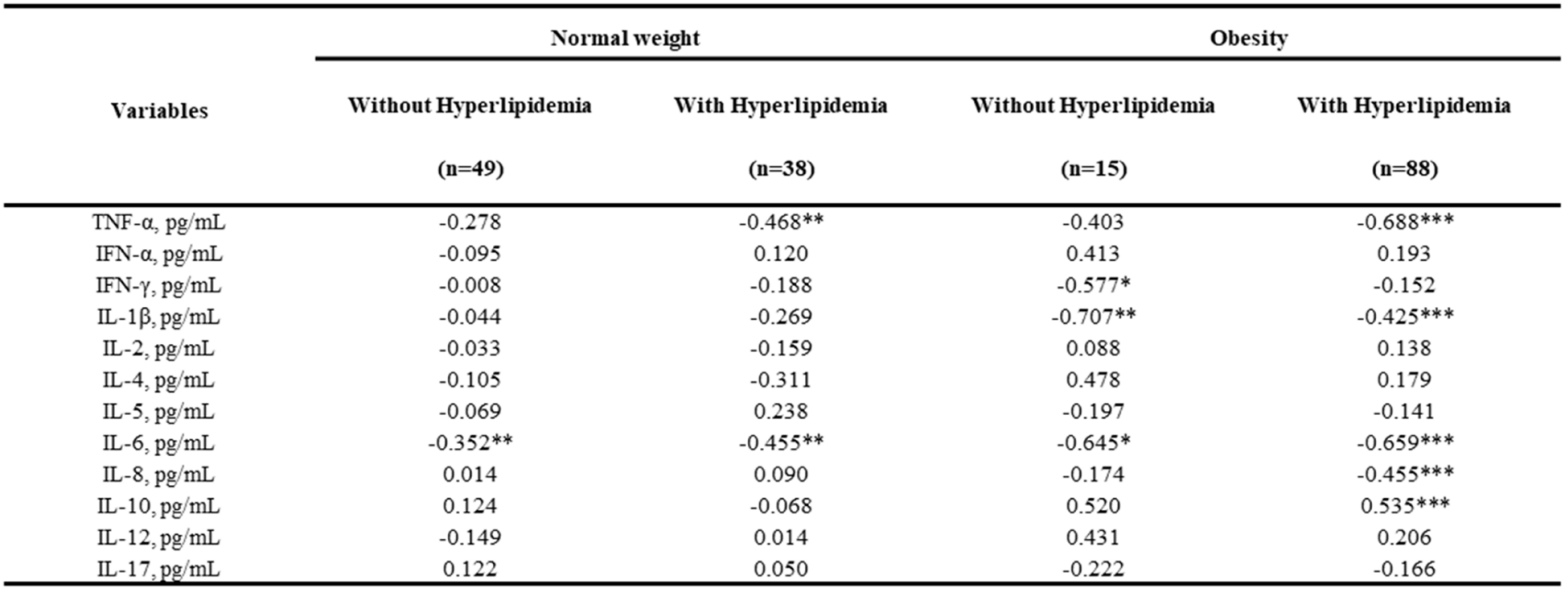
Correlations between inflammatory cytokines and bmd among obese subgroups stratified by hyperlipidemia

Models were adjusted for sex, age, BMI, smoking and drinking alcohol history, hypertension, and diabetes

### Mediating role of inflammatory cytokines in the lipid Metabolism-Bone density relationship

Subsequently, the study involved the execution of mediation analysis to further explore the interrelationships among inflammatory cytokines, lipid metabolism, and OP in obese individuals. As shown in Table 7, TNF-α and IL-6 acted as dominant mediators of the TC-BMD association, accounting for 41.91% and 33.20% of the total effect, respectively. Partial mediation of the TG–BMD relationship was attributed to IL-10 and IL-1β, which explained 10.99% and 7.09% of the mediation effect, respectively. Regarding HDL-C, IL-6 (37.38%) and TNF-α (28.76%) demonstrated the highest mediation proportions. Additionally, in the ApoB pathway, TNF-α exhibited exceptionally strong mediation (61.09%), followed by IL-6 (39.38%). These findings suggest that inflammatory cytokines serve as biological intermediaries, mechanistically linking obesity-associated lipid dysregulation with elevated OP risk.

**Table 7:**
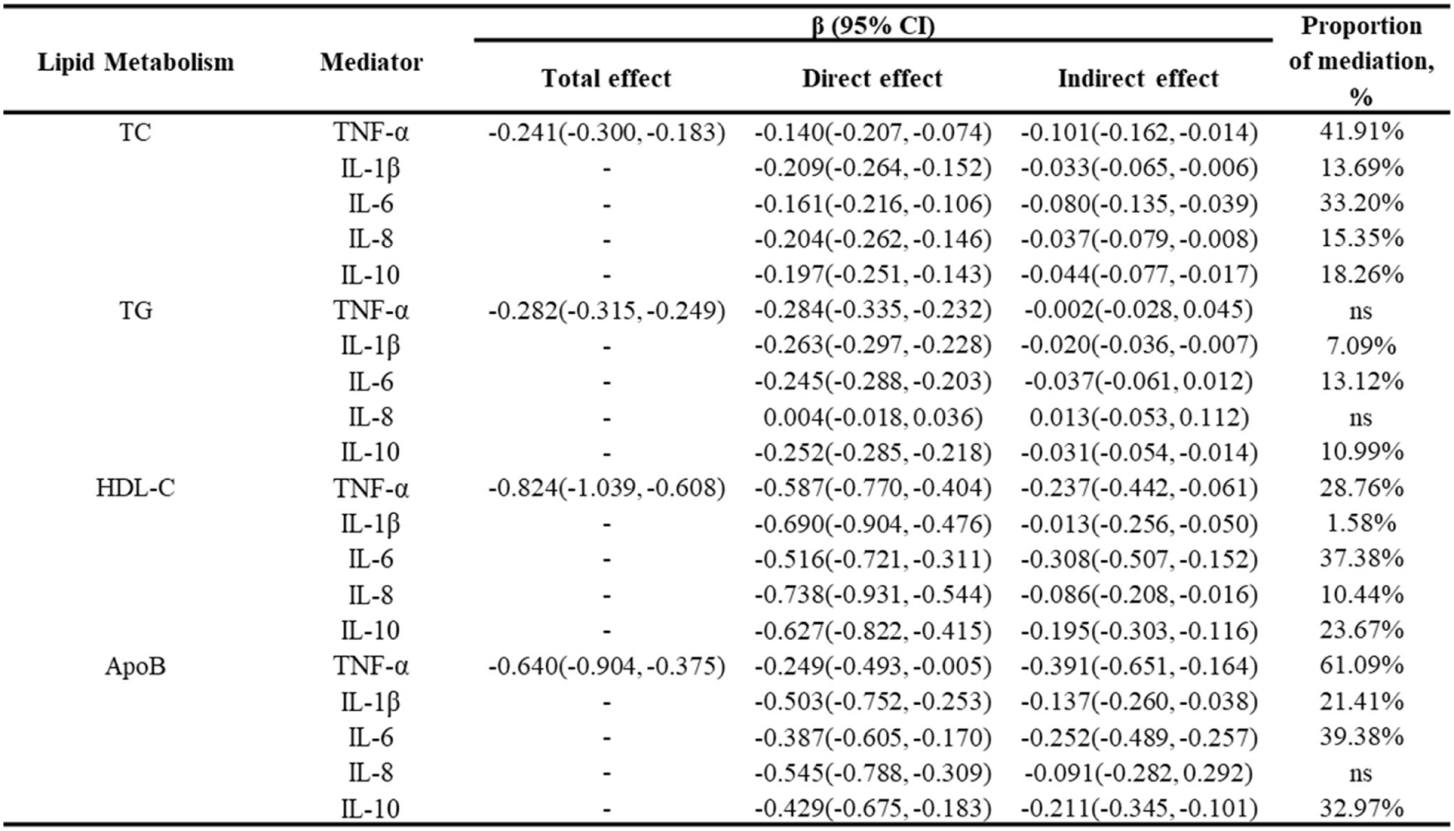
Mediation effect of inflammatory cytokines on the association between lipid metabolism and BMD

## Discussion

This study offers an extensive retrospective investigation into the association between obesity and the risk factors for OP. We found that abnormal lipid metabolism, indicated by levels of TC, TG, HDL-L, and ApoB, was significantly correlated with enhanced BMD loss in individuals with obesity. Additionally, obese individuals with hyperlipemia had notably elevated levels of inflammatory cytokines and decreased levels of BMD compared to normal weight individuals. Importantly, the mediation analysis suggested that TNF-α, IL-1β, IL-6, IL-8, and IL-10 partially mediated the association between lipid metabolism dysfunction and the heightened risk of OP in obese patients.

In the past decade, the prevalence of obesity has risen to pose a serious health issue across the globe, pathologically characterized by the convergence of adipose tissue accumulation and dysregulated lipid metabolism [32]. Due to its established association with metabolic imbalance, impaired immune function, and inflammation, elevated BMI is epidemiologically associated with several chronic diseases, such as diabetes, hypertension, atherosclerosis, fatty liver disease, malignancies, and musculoskeletal disorders [33]. Earlier studies have shown inconsistent results concerning the relationship among obesity, the risk of OP and fractures. For instance, Lin’s research on 1,007 Taiwanese postmenopausal women revealed a phenomenon that sarcopenic obesity was linked to a reduced risk of OP and significantly increased the likelihood of fragility fractures [34]. Qi et al. reported that overweight/obesity suggested higher BMD, whereas underweight individuals showed a significantly reduced BMD [35]. In contrast, studies have also reported that excessive lipid accumulation in the blood and bone can lead to dyslipidemia, which can harm osteoblasts and leads to OP. Previous research reported an inverse correlation between TG levels and BMD in the elderly population [36]. Consistent with this result, Cao et al. demonstrated that elevated TC exhibited a significant negative correlation with total BMD, particularly pronounced in young adults aged 20–29 years [37]. Sun et al. further demonstrated that the connection between visceral fat and femoral BMD followed an inverted U-shaped pattern, suggesting there may be optimal fat thresholds for maintaining bone health [20].

In this study, our data revealed significantly lower BMD in obese versus non-obese OP patients and identified TG, TC, HDL-C, and ApoB as significant predictors of accelerated BMD loss in obesity. This finding contradicts classical views that obesity protects bone health via mechanical loading. This paradox may be explained by distinguishing between metabolically healthy and dysfunctional obesity. While excessively accumulated adipose tissue may support bone formation through mechanical stress and estrogen release, visceral adiposity drives dyslipidemia, pro-inflammatory cytokine secretion and oxidative stress, collectively promoting bone resorption [11, 38, 39]. Recent studies also demonstrate this opinion. Sun et al. reported an inverted U-shaped relationship between visceral fat and BMD, suggesting detrimental effects beyond an optimal threshold [20]. Similarly, Lin et al. observed that sarcopenic obesity increased fracture risk despite higher BMI [34]. Our findings align with these models, indicating that in obesity-related OP, metabolic dysfunction overrides mechanical benefits, leading to net bone loss.

According to the recent studies, the impact of obesity on OP is further complicated by the inflammatory environment associated with dysregulated lipid metabolism [38, 40]. Obesity results an excessive accumulation of lipids in bone marrow and bone tissue, triggering inflammation and disrupting bone metabolism, which ultimately contributes to bone loss and the development of OP [14, 41]. Specifically, excessive accumulations of lipids, particularly TC, TG, and their metabolites, activate inflammation in osteoblasts and suppress osteoblasts differentiation and mineralization. These pathophysiological disruptions subsequently manifest as trabecular bone loss and cortical bone thinning, adversely impacting bone microstructure and BMD [42]. In addition, adipose tissue, now understood as a significant site of lipid metabolism in bone, secretes pro-inflammatory cytokines and adipokines that impair bone homeostasis by stimulating osteoclastogenesis and consequently elevating bone resorption, ultimately reducing BMD [38]. These findings substantiate that chronic inflammation triggered by dyslipidemia constitutes a central pathological mechanism underlying obesity-related osteoporosis. Based on the retrospective clinical study of postmenopausal women, Jin et al. demonstrates significant correlations between inflammatory indicators, such as IL-6, and osteoporosis development, supporting the critical role of inflammation in BMD loss [43]. Some researchers found that elevated serum levels of specific pro-inflammatory cytokines, particularly IL-8, IL-17, and IL-22, are significantly positively correlated with osteoporosis development in postmenopausal women [44]. Consistent with previous researches, our results reveal that abnormal lipid metabolism plays a crucial role in obesity-related osteoporosis by regulating the release of inflammatory cytokines. Critically, in obese patients with hyperlipidemia, pro-inflammatory cytokines (TNF-α, IL-1β, IL-6 and IL-8) emerged as potent negative predictors of BMD, whereas IL-10 positively correlated with bone density. These data establish hyperlipidemia as a key accelerator of inflammation-driven bone catabolism specifically in the obese population. Given the essential contribution of lipid metabolism and chronic inflammation to bone homeostasis, elucidating key inflammatory mediators linking obesity to osteoporosis is imperative. Investigating this crosstalk holds substantial clinical significance for identifying novel therapeutic targets.

However, our mediation analysis identified TNF-α and IL-6 as significant mediators in the lipid-bone relationship. We also acknowledge that dyslipidemia-induced bone loss operates through multifactorial pathways beyond inflammation. For instance, oxidative stress represents a plausible alternative mediator, as lipid peroxidation products accumulate in obesity and directly impair osteoblast function while promoting osteoclastogenesis via RANKL upregulation [45]. Hormonal alterations further link obesity to osteoporosis. Leptin resistance in adipose tissue may reduce osteoblastic activity, while adiponectin deficiency diminishes bone formation [46, 47]. Sex hormones (estrogen/testosterone) and gut-derived hormones (GLP-1) could also mediate this pathway [46, 48]. Although our study did not measure these biomarkers, clinical evidence suggests their involvement in obesity-related bone loss. In our study, while TNF-α and IL-6 explain considerable variance, unmeasured mediators like oxidative molecules or adipokines likely contribute to residual effects. Future studies quantifying oxidative markers, adipokines, and bone-related hormones are needed to fully dissect machinery connecting dyslipidemia and OP.

In this research, we focus on the role of inflammatory cytokines as mediators in the connection between obesity and OP. This pathophysiological connection has been systematically reported in other obesity-associated diseases, including cardiovascular disease, asthma, diabetes and etc [21, 28, 30]. For instance, Alizadeh et al. reported that dysregulated inflammatory factors, especially CRP, IL-1β and IL-10, is an intermediary factor in the associations between obesity and atherosclerosis [49]. Ingram’s study identified that leptin, an obesity-associated adipokine, synergizes with IL-13 to directly mediate pulmonary eosinophilia in obesity-associated type 2 asthma [50]. In summary, these results reinforce the key role of pro-inflammatory cytokines in facilitating lipid dysmetabolism as obesity triggers disease progression. In this study, mediation analysis further confirmed that five inflammatory cytokines (TNF-α, IL-1β, IL-6, IL-8, and IL-10) significantly mediate the link between dysregulated lipid metabolism and reduced BMD in individuals with obesity. Based on these results, targeted regulation of these cytokines might serve as a potential strategy for therapy for preventing obesity-related OP.

While evidence from our investigation indicates inflammatory cytokines potentially mediate interactions connecting adipose tissue accumulation to reduced BMD, several limitations must be acknowledged. Firstly, the retrospective single-center design incorporates a limited small cohort, potentially affecting the statistical power and might accounting for some conclusion lacked statistical significance. Secondly, due to the practical constraints of patient-derived clinical data collection, we have only measured circulating lipid and inflammatory cytokines levels of participants at a single time point, which might obscure genuine temporal variations in how adiposity impacts OP and fracture risk. Thirdly and most importantly, despite our statistical models revealing associations between dyslipidemia (TC, TG, HDL-C) and BMD mediated by inflammatory markers, the retrospective observational design precludes causal inference. These associations may reflect bidirectional relationships or unmeasured confounders. Furthermore, although strict pre-enrollment screening excluded participants taking hormones, statins, and anti-osteoporosis medications and accounted for physical activity, residual confounding remains likely. Importantly, unmeasured variables, including longitudinal medication histories, granular comorbidity data, and formalized exercise metrics, might not solely act as confounders but could represent additional mediators within the adipose-tissue-bone axis, potentially altering the interpretation of the identified pathways. Consequently, future clinical investigations should adopt prospective longitudinal designs with serial biomarker measurements. This approach is essential to verify causality, elucidate dynamic mechanisms, elevate evidence quality, and inform therapeutic strategies.

## Conclusion

This study established notable associations between blood lipid, inflammatory cytokines, and BMD, presenting them as potential markers for OP occurrence in patients with obesity. Specifically, dysregulated blood lipid metabolism promotes accelerated BMD loss in obese individuals by stimulating inflammatory cytokine secretion, thereby exacerbating OP pathogenesis. This mechanistic link identifies lipid-inflammatory pathways as novel targets for OP diagnostics and therapeutic intervention, particularly among obese populations. Nevertheless, considering the limitations of this study, future research should aim to further uncover the specific mechanisms of the lipid–inflammation–bone axis and create optimal therapeutic strategies, including TNF-α/IL-6-inhibiting biologics for high-risk patients, statins for dyslipidemia control, and anti-inflammatory diets to concurrently mitigate lipid dysregulation and inflammation, thereby advancing the prevention and management of OP and fractures.

## Data Availability

The data used to support the findings of this study are available from the corresponding author upon reasonable request (Corresponding email; Xiexing Wu, wuxiexing@163.com; Xiuqiang Lv, 824814363@qq.com).

## Abbreviations

TC: Total cholesterol
TG: Triglycerides
HDL-C: High-density lipoprotein cholesterol
LDL-C: Low-density lipoprotein cholesterol
ApoA: Apolipoprotein A
ApoB: Apolipoprotein B
Lp(a): Lipoprotein(a)
TNF: Tumor necrosis factor
IFN: Interferon
IL: Interleukin
